# Free and open-source QSAR-ready workflow for automated standardization of chemical structures in support of QSAR modeling

**DOI:** 10.1186/s13321-024-00814-3

**Published:** 2024-02-20

**Authors:** Kamel Mansouri, José T. Moreira-Filho, Charles N. Lowe, Nathaniel Charest, Todd Martin, Valery Tkachenko, Richard Judson, Mike Conway, Nicole C. Kleinstreuer, Antony J. Williams

**Affiliations:** 1 National Toxicology Program Interagency Center for the Evaluation of Alternative Toxicological Methods, National Institute of Environmental Health Sciences, Research Triangle Park, NC 27709 USA; 2 Center for Computational Toxicology and Exposure, Office of Research and Development, U.S. Environmental Protection Agency, Research Triangle Park, NC 27711 USA; 3 ScienceDataExperts LLC, Rockville, MD 20850 USA; 4 National Institute of Environmental Health Sciences, Research Triangle Park, NC 27709 USA

**Keywords:** QSAR-ready, Standardization, Molecular structures, InChI, KNIME

## Abstract

The rapid increase of publicly available chemical structures and associated experimental data presents a valuable opportunity to build robust QSAR models for applications in different fields. However, the common concern is the quality of both the chemical structure information and associated experimental data. This is especially true when those data are collected from multiple sources as chemical substance mappings can contain many duplicate structures and molecular inconsistencies. Such issues can impact the resulting molecular descriptors and their mappings to experimental data and, subsequently, the quality of the derived models in terms of accuracy, repeatability, and reliability. Herein we describe the development of an automated workflow to standardize chemical structures according to a set of standard rules and generate two and/or three-dimensional “QSAR-ready” forms prior to the calculation of molecular descriptors. The workflow was designed in the KNIME workflow environment and consists of three high-level steps. First, a structure encoding is read, and then the resulting in-memory representation is cross-referenced with any existing identifiers for consistency. Finally, the structure is standardized using a series of operations including desalting, stripping of stereochemistry (for two-dimensional structures), standardization of tautomers and nitro groups, valence correction, neutralization when possible, and then removal of duplicates. This workflow was initially developed to support collaborative modeling QSAR projects to ensure consistency of the results from the different participants. It was then updated and generalized for other modeling applications. This included modification of the “QSAR-ready” workflow to generate “MS-ready structures” to support the generation of substance mappings and searches for software applications related to non-targeted analysis mass spectrometry. Both QSAR and MS-ready workflows are freely available in KNIME, via standalone versions on GitHub, and as docker container resources for the scientific community. *Scientific contribution*: This work pioneers an automated workflow in KNIME, systematically standardizing chemical structures to ensure their readiness for QSAR modeling and broader scientific applications. By addressing data quality concerns through desalting, stereochemistry stripping, and normalization, it optimizes molecular descriptors' accuracy and reliability. The freely available resources in KNIME, GitHub, and docker containers democratize access, benefiting collaborative research and advancing diverse modeling endeavors in chemistry and mass spectrometry.

**Supplementary Information:**

The online version contains supplementary material available at 10.1186/s13321-024-00814-3.

## Introduction

The need for the investigation of chemicals’ physicochemical properties and biological activities, both in vitro and in vivo, commonly requires the use of computational predictive approaches. In silico methods such as quantitative structure–activity/property relationship (QSAR/QSPR) models represent important tools for virtual screening, data gap-filling and prioritization for testing. It also facilitates the initial research and development optimization of desirable molecular characteristics. Such models are trained on experimental data and rely entirely upon molecular structure representations to generate predictions from the learned relationships between structure and activity/property.

The ever-expanding experimental datasets available from public sources allow for rapid development and improvement of predictive models for different biological and toxicological endpoints as well as physicochemical properties. However, when transforming molecular graphs and their associated data into predictions and insights, the quality of the input data sets an upper limit on the quality of the output. Thus, the predictivity and accuracy of the developed models highly depends upon the quality of the training data. Several methods have been developed and applied to curate experimental data using natural language processing techniques, as well as statistical methods for outlier detection, to detect and possibly correct reporting errors and unit conversion mistakes [1–3]. The associated molecular graphs, however, have historically not received the same level of attention. Although machine-based molecular encodings have been in use for over six decades, relatively little attention has been directed to curate the chemical structure representations that are the basis for training the models and applying them to provide predictions on new chemicals. Some highlighted studies include our own previous works in this area (2016) and those of Fourches et al. (2010), Williams and Ekins (2011), and recently Lowe et al. (2023) [4–9]. However, failure to address this problem has negative consequences not only on property prediction and classification during training and use, but also affects registration and deduplication as well as similarity and substructure searches within databases.

The conceptual basis of QSAR/QSPR and related approaches is the congenericity principle: the assumption that similar structures are associated with similar properties and/or biological activities. Thus, the unknown properties of a given chemical can be inferred from compounds with known experimental responses based on molecular structure. Predictive models are trained by applying machine learning algorithms relating specific structural features of chemicals to their associated experimental responses. However, this procedure usually requires a prior step to derive the appropriate structural features from the molecular representation which mainly depends on its intrinsic nature. This step consists of retrieving the corresponding information encoded in the molecular structures then converting it to an array of discrete or continuous numbers called molecular descriptors.

The process of generating molecular descriptors used to establish the correct relationships between the training structures and the experimentally demonstrated properties is based on the molecular representation. However, a molecular structure has no unique representation. Instead, several models exist depending on the theoretical approach adopted and the degree of approximation. The complexity of a molecular structure relates to the fact that most of its properties cannot be derived from only considering its single atoms; rather, it is a holistic system that depends on the atomic connections and interactions. Any change in these parameters can alter a molecular descriptor value. Thus, standardization of the molecular representation rules is important to achieve consistency in terms of descriptor values between the training step and any future model application to generate predictions.

These standardization approaches deal with issues such as tautomerization, standardization of functional groups (e.g., nitro group and azo group representations), salt handling, and other standardization concerns to bring structure sets into coherence under a defined set of rules. Despite their importance to the outcomes of QSAR/QSPR modeling, there was little consideration of general guidelines or protocols regarding standardization or curation of molecular graph encodings in the literature until recently. The first real mention of chemical structure errors in QSAR datasets is a 2008 study by Young et al. [10]. In 2010, a pivotal review by Fourches et al. demonstrates the non-negligible effects of molecular graph errors on the performance of QSAR models and their accuracy [6]. Until these two publications, the problem was only treated tangentially, as stated in the Fourches et al. review.

Since then, several reports and tools have been published reflecting the burst of publicly available datasets. These data are commonly assembled from historical collections and are consequently affected by a significant lack of molecular graph curation. This ‘snowballing’ problem emphasizes the need for community guidelines. While there are vendor-provided solutions such as MDL Cheshire (https://med.stanford.edu/content/dam/sm/htbc/documents/mdl_cheshire_ds.pdf), and ChemAxon (https://docs.chemaxon.com/display/docs/standardization-in-step-by-step.md), these approaches have their benefits locked behind potentially insurmountable barriers for QSAR/QSPR modelers without the means to access them.

Public access tools have only been available in recent years. The first noteworthy online tool was the Chemical Validation and Standardization Platform (CVSP) delivered in 2015 by the ChemSpider team [11]. CVSP was designed to help with the detection of issues in molecular representations using a predefined dictionary of molecular patterns, the ability to define and upload custom sets of standardization operations, alongside the option to perform manual review. Inspired by the CVSP platform, and the concerns outlined in Fourches et al., an early version of the QSAR-ready standardization workflow, subject of the current manuscript, is first mentioned in 2016 by Mansouri et al. [4]. Several other tools have since appeared in the literature describing other standardization approaches and these include MolVS in 2017, PubChem standardization in 2018, an RDKit chemical structure curation pipeline (from the ChEMBL group) in 2020, and IBM’s standardization library in 2022 [12–18].

This publication describes the development of the QSAR-ready standardization workflow and its automated process of generating curated standardized structures for the molecular descriptors calculation step of modeling. This standardization can be performed as a desktop application and does not require submission to any web-based application. The first version of the application was developed in 2015 at the U.S. Environmental Protection Agency (EPA). The resulting software product provided regulatory scientists, students, and researchers with the ability to effectively process multiple in silico data streams in support of various regulatory decision frameworks.

The workflow was designed using the Konstanz Information Miner platform commonly referred to as KNIME [19]. The first version of the workflow, developed in 2014, was used to standardize thousands of chemical structures for the international collaborative estrogen receptor activity prediction project (CERAPP), led by the EPA and published in 2016, providing the workflow as supplemental material [4]. This initial workflow was incrementally improved over time and continues to be used to standardize molecular structures for the interagency suite of QSAR models known as OPERA [20, 21]. As a result of the inherent simplicity and flexibility of the workflow, it was adapted to standardize structures to support mass spectrometry (MS), specifically to support the non-targeted analysis (NTA) with high-resolution mass spectrometry (HRMS) research efforts at the US-EPA [22].

The modified “MS-Ready” workflow has also been used to process the entire contents of the DSSTox database to produce the “MS-ready structures” that are used on the EPA’s CompTox Chemicals Dashboard (from hereon, the Dashboard) as the basis of the Linked Substances capability [23, 24]. More recently, the QSAR-Ready workflow proved to be essential to the global collaborative modeling projects to predict both androgen receptor activity (CoMPARA) and acute oral toxicity (CATMoS) [25, 26]. Additionally, it was used to standardize the QSAR-ready structures also available on the Dashboard via the batch search and national toxicology program interagency center for the evaluation of alternative toxicological methods (NICEATM) integrated chemical environment (ICE) [24, 27, 28].

## Materials and methods

### Background

Analysis of molecular structure data, typically graphs, to generate insights or predictions is foundational to computational chemistry and cheminformatics. In these research areas the performance of the modeling outputs is set by the quality of the input. In this context, the quality of the empirical input data has been the subject of much study and is increasingly well defined [29]. However, when considering the associated molecular graphs, the question of what aspects of representation should be fixed or rejected and under what conditions is of utmost consideration. These issues are generally addressed in machine learning literature. However, attention within QSAR/QSPR and as representation pertains to chemical data and inputs remains under-inspected [30].

One of the first big leaps that contributed to modern computational chemistry was the storage of two-dimensional structures by the IBM 704 computer system [31]. This allowed chemists to draw any structural fragment or moiety, search the stored files, and have the computer print it in a format that did not require translating or decoding. Since then, the rapid evolution of computer science technologies has brought revolutionary advancements in modern data analytics. Presently, it is possible to analyze enormous streams of chemistry-related data in depth and handle a multitude of parameters simultaneously. In terms of a standardization workflow, this allows not only for the detection and removal of erroneous structural representations, but also for the repair and harmonization of data with the application of specific, predefined rules.

For every standardization process reported in the literature to date, three main steps are employed which aim to reduce factors that interfere with extracting accurate predictions and insights from molecular graphs [6, 10, 11, 32].

The first step is always related to parsing the input file containing the chemical structure(s) and checking its validity. There are multiple file formats for storing chemical structures, but the most commonly used are the MOL file (originally developed by MDL Information Systems and now managed by Dassault Systems) storing a single chemical in multiline string format, the structure data file (SDF) composed of multiple structures concatenated in MOL format, and the simplified molecular-input line-entry system (SMILES) that defines a structure in a single line string format initially developed at the US EPA, but managed and enhanced by Daylight [33–36].

Each of the file formats has its own syntactical rules for encoding atom and bond types as well as the two- and three-dimensional information and coordinates. Violations of these rules usually lead to parsing errors or warnings. While some violations can be programmatically corrected, others will corrupt the structural integrity of the chemical representation and lead to a complete parsing failure. This speaks to the importance of establishing standardized workflows compatible with common molecular file types to prevent automated pipelines from failing due to violations of established encoding standards.

The second step assesses the consistency of the input file and depends upon the presence of additional information about the chemical structure. For example, MOL and SDF files may include several fields storing information such as systematic names, international chemical identifiers (InChI), Chemical Abstracts Service registry number (CASRN^®^), among others. The role of this step is to check how the molecular graph information reflects the known features implied by those identifiers. The consistency of each file entry can be ranked and used to determine if and how the validity of the input can be restored in case of a parsing error or warning [5].

The third and main step is what is usually intended by the standardization process. It consists of checking if the molecular graph breaks any commonly known or subjectively predefined rules for representation form, style, or semantics.

### Standardization process

The complexity of the workflow increases dramatically over the mentioned steps. The first is relatively simple since any cheminformatics reader tool should report format-related parsing errors that can be directed towards review and repair or removal of the entry. The degree of importance and complexity of the second step depend on the nature and number of what identifiers are available and whether they are internal to the input or external to it. The third step is the most complex but also the most critical. Its rules and operations may vary by organization, team, and project, and/or dataset.

However, once well-defined and self-consistent rule sets are defined, the rules support the implicit requirement for standardization on any new dataset, including chemical datasets generated outside the rule-setting organization. As would be expected, some dataset-specific exceptions may apply to certain rules and there is not a “one-size-fits-all” guide to standardization.

Thus, the objective of exhaustively cataloging the different operations is to provide the richest possible set of options to generate structures for one type of application and, in this case, to generate “QSAR-ready” structures suitable for descriptor calculation and model building. A secondary goal is to make the set of rules as flexible as possible for expert curators to combine and select rules as needed to adapt for other applications. An example of this, as mentioned previously, the “MS-ready” standardization workflow to support specific searches supporting mass-spectrometry as detailed in McEachran et al. [22].

In general, a standardization rule is a two-step sequence that checks for the presence of a specific feature and then prescribes one or more graph transformations to remedy the potential issue. The standardization process involves an ordered specification of these rules, bearing in mind that some steps are order-dependent and lead to a different outcome if commuted (Additional file 1: S1) [37].

The proposed process in this work is customizable and consists of the following procedures:

- A broad number of checks and remedies for valency including hypervalent nitrogen, sulfur, phosphorous, N-oxides, nitro compounds, sulfoxides, sulfones, phosphates, etc.
- Filter metals, inorganics, and organometallics.
- Filter ambiguous structures that passed the initial parsing such as Markush structures and R-groups.
- Stripping of salts and solvents using a block list (integrated in the workflow). The inorganic and organic fragments that are in the list of known salts and solvents are carried through separately for consideration when they may be included in modeling efforts in, for example, melting point or partition coefficients.
- Filter organic mixtures. Any substance with unconnected organic fragments that are not identified in the previous steps as a known salts or solvents, are filtered out as mixtures which does not pass standardization.
- Flatten stereochemistry for two-dimensional output and carry the stripped information for the three-dimensional output, if requested.
- Virtualize explicit hydrogens, specifically to support tautomers (make nonstereogenic explicitly represented hydrogens implicit).
- A series of bond/group standardization and neutralization rules when required:
  - Protonate or deprotonate the parent ion when a salt/counterion is removed.
  - Zwitterion to covalent bond.
  - Standardize nitro mesomers.
  - Tautomerize groups based on a list of rules and transformations provided in a reaction format.
  - Ambiguous double bond conformation to crossed double bond.
- Switch rings to aromatic, according to Hückel’s rule, or Kekulé as requested [38, 39].
- Sanitize atomic coordinates.
- Generate InChI and InChI keys.
- Collapse duplicates based on the IUPAC (International Union of Pure and Applied Chemistry) InChI (International Chemical Identifier) codes because these are unequivocal identifiers, if requested.
- Filter based on size and shape, if requested (number of heavy atoms, molecular weight, nano material shapes).
- Generate canonical SMILES.
- A series of standardization rules for three-dimensional structures, if requested, including:
  - Add explicit hydrogen atoms.
  - Generate three-dimensional coordinates.
  - Optimize conformation geometry.
- Output format containing the following data fields:
  - QSAR-ready SDF file (both 2D and 3D files available).
  - QSAR-ready SMILES file: canonical QSAR-ready SMILES and the provided main entry identifier.
  - Stripped salt/solvent information.
  - Filtered out entries with a field specifying the reason: failed parsing, mixtures, inorganics, organometallics, size (based on the molecular weight and number of heavy atoms, nano-shaped molecules (based on a ratio of number of bonds and number of heavy atoms).
  - Summary CSV file of the processed QSAR-ready structures including identifiers, InChI/InChIkeys, canonical SMILES, and additional processing details.

By far the most complicated step in this process is the application of tautomerization and neutralization rules [40]. In this work, this step includes more than a hundred transformations that are applied individually and turned on or off by expert users (Additional file 2). Most of these transformations were adapted from the earlier work of Sitzmann et al. [41].

### Workflow development under KNIME

Software to manage a QSAR-ready standardization workflow of the complexity described above would be a challenging task to develop from scratch using a programming language or custom scripts. For the widest accessibility and adoption, fully mature standardization software requires a user interface to navigate and customize the different processes, in addition to the high level of flexibility necessary to navigate through the various data processing steps. It further needs augmentation with titles, annotations, and descriptions to guide users through the process.

The KNIME analytics platform is a free and open source software platform that fits these requirements by providing a flexible environment that can be extended with third party tool integrations and allows for step-by-step documentation in great detail, all by graphical interface that can be customized as needed [19, 42]. KNIME’s modular setup enables users to assemble and modify the analysis flow through a visual interface using standardized building blocks called nodes. These nodes are connected by pipes that transfer data and/or instructions and can be manipulated with minimal to no programming experience. KNIME boasts a broad range of capabilities allowing users to develop workflows for different fields, but the platform has a specific focus on cheminformatics and bioinformatics with a thriving community continuously developing and updating nodes. In addition to the ability to execute different external free/open source and commercial/proprietary software with no need for data formatting or calling for dependencies, KNIME also integrates multiple programming languages allowing users to run existing scripts. The resulting workflows developed in KNIME can be executed either locally on a desktop using the platform’s graphical or command line interfaces, as well as using the KNIME server WebPortal that offers a user-friendly web application experience. Unlike the desktop version of KNIME that is downloadable for free, the server version requires a license. The workflows can also be shared in various ways to enable collaboration and reusability among users, which is crucial for effective data analysis. The KNIME Server offers a programmable interface, known as the KNIME Server API, that allows developers to interact with the platform. This API consists of RESTful web services that enable users to execute workflows, manage workflows and jobs, retrieve workflow and job information, access data, and more. With the KNIME Server API, users can automate, integrate, and customize workflows and services provided by the server. The API supports standard HTTP methods (GET, POST, PUT, DELETE) and facilitates data exchange in various formats like JSON and XML. Additionally, it provides comprehensive error messages and status codes for effective error handling and troubleshooting. By utilizing the KNIME Server API, developers can seamlessly integrate KNIME Server functionality into their own applications, automate workflow and job management, process data, and create tailored solutions that interact with the server [43, 44].

The KNIME platform is particularly well-suited for the QSAR-ready workflow due to its software stability and backward compatibility. Unlike other software, old nodes in KNIME are not removed but rather deprecated, allowing workflows created using previous versions to be executed with the same level of reliability.

Since the workflows can get complicated when performing multiple tasks (e.g., database queries, data analysis, modeling, visualization), KNIME provides the option to annotate the different steps and add documentation when necessary. This is important for sharing and collaboration among teams of developers or between the developers and users.

### Automation and input/output interfaces

To make the most of the features that the KNIME platform offers in terms of automation, user-friendliness, sharing, and flexibility, a QSAR-ready standardization workflow was developed and equipped with all the required components. In addition to the standard possibility to configure nodes independently and run the workflow step-by-step, the workflow includes different components that allow the user to configure the entire workflow at once, and then run it with the push of a button.

This is made possible with an input window that prompts the user to browse for the input file (MOL, SDF or SMILES) and select the different options for running the workflow (i.e., aromaticity, 2D/3D, size limit). These parameters are carried through the entire workflow as local “flow variables” to automatically configure the different nodes without further user interaction.

Although the workflow is thoroughly documented at the node level and the different steps annotated, the user interface remains a much easier way to run the workflow and is extremely useful given this workflow contains hundreds of nodes and multiple layers of encapsulation. The flow variables carry the input file name and path, automatically name the output files accordingly, and exports them to the same input path.

In addition to the flow variables, the workflow is established with global variables that allow the user to programmatically interact with and run the workflow in a batch mode behind the scenes without opening the KNIME platform graphical interface. This option is useful for running a KNIME workflow via command line or for integration with other tools running outside the KNIME environment.

For example, this option was used to embed the QSAR-ready workflow within the OPERA suite [21]. OPERA is a free and open-source data and QSAR modeling software described in detail later in the Applications section. Since all OPERA models employ this same QSAR-ready workflow to standardize the training set chemical structures prior to modeling, it is crucial to use the same process to standardize user input chemical structures during prediction. The general command to run the workflow in command line is as follows:

*“knime-nosplash-application org.knime.product.KNIME_BATCH_APPLICATION [options]”*. For additional information about the structure of the batch mode commands, refer to the KNIME website (https://www.knime.com/).

Setting up the workflow with flow and global variables is necessary before deploying to the KNIME server. These variables specify the choices of the user through the different steps of the standardization process. However, for the workflow to be server ready, it is required to include specific components encapsulating relevant interactive widgets (https://hub.knime.com/knime/extensions/org.knime.features.js.views/latest) into “quickform” metanodes that display the choices on the KNIME WebPortal and allows for interactivity with users. This step is called “workflow abstraction” and is necessary to display the different interactive webpages on the WebPortal. The latest version of the QSAR-ready workflow (as of August 2023) includes two levels of interaction with a user-dependent input format that can be either an uploaded file (MOL, SDF, SMILES), or direct input structures on the WebPortal, by either pasting SMILES or drawing the structures using the implemented rendering tool. At the end of the execution of the workflow, the resulting structures are displayed on the WebPortal, and output files provided for download.

The KNIME server version of the workflow can also be accessed and executed via the server’s RESTful API. The chemical structure information (MOL, SDF, SMILES) can be sent and received in a wrapped JSON format through standard HTTP methods such as GET, POST, PUT, and DELETE [43, 44].

### Sharing options

KNIME provides several ways for users to share their workflows with others, including the KNIME Hub, GitHub, Docker containers, and KNIME Server. These platforms enable collaboration and reusability among users, streamline data analysis workflows, and make it easier for organizations to manage their data science resources.

One way to share KNIME workflows is through the KNIME Hub, which is a community-driven platform that allows users to share, discover, and collaborate on workflows. Users can upload their workflows to the platform and make them either public or private. Other users can then download and use these workflows or contribute to them by adding comments or making improvements. The QSAR-ready workflow is available on the KNIME Hub at: https://kni.me/w/5TRvnGfMJsgTkcZu.

Users can also share KNIME workflows through GitHub, a web-based platform for software version control and collaboration. This enables multiple users to work on the same workflow simultaneously and track changes over time. GitHub also supports integration with other tools such as continuous integration and delivery tools. The QSAR-ready workflow is available on the NIEHS GitHub repository: https://github.com/NIEHS/QSAR-ready.

Another way to share KNIME workflows is through Docker containers. Docker is a platform for developing, shipping, and running applications using containers. By creating a container image that includes the KNIME workflow and all the dependencies required to run it, users can share their workflows with others and run them on any machine that supports Docker, regardless of the underlying operating system or hardware. The QSAR-ready workflow is available as a docker container at: https://hub.docker.com/r/kamelmansouri/qsar-ready. The docker file is available on GitHub at: https://github.com/kmansouri/QSAR-ready which is built on a KNIME base image developed by: https://github.com/ibisba/knime-workflow-base.

Finally, KNIME Server provides a web-based platform for centralized management of workflows, data, and users. By deploying their workflows to the server, users can share them with other users in their organization, run them remotely, schedule them to run at specific times, and monitor their execution. The QSAR-ready workflow is available on NIEHS’ KNIME server WebPortal at: knime.niehs.nih.gov/knime**/.**

## Results and discussion

### Workflow overview

**Fig. 1:**
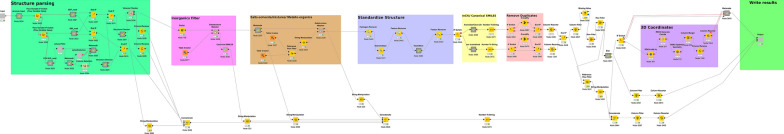
KNIME QSAR-ready chemical structure standardization workflow organized by sections of the process

The QSAR-ready workflow described above, and illustrated in Fig. 1, comprises eight major steps: (1) input and structure parsing, (2) inorganics filter, (3) salts, counterions and mixtures processing, (4) structure standardization, (5) ring processing, (6) duplicates processing, (7) 3-dimensional structure processing, (8) output. Each of these steps is annotated on the workflow with a different color and consists of several sub-steps executed by various nodes and metanodes. Additional notes and documentation are available in the metanodes to provide more detailed explanations of the process, particularly for advanced KNIME users and workflow developers who wish to modify or reconfigure the workflow. These notes and documentation are only visible when accessing the workflow in the KNIME desktop user interface. Alternatively, the KNIME server WebPortal provides a distinct form of documentation that is designed to guide users through the different interactive steps requiring their input.

#### Input and workflow execution

**Fig. 2:**
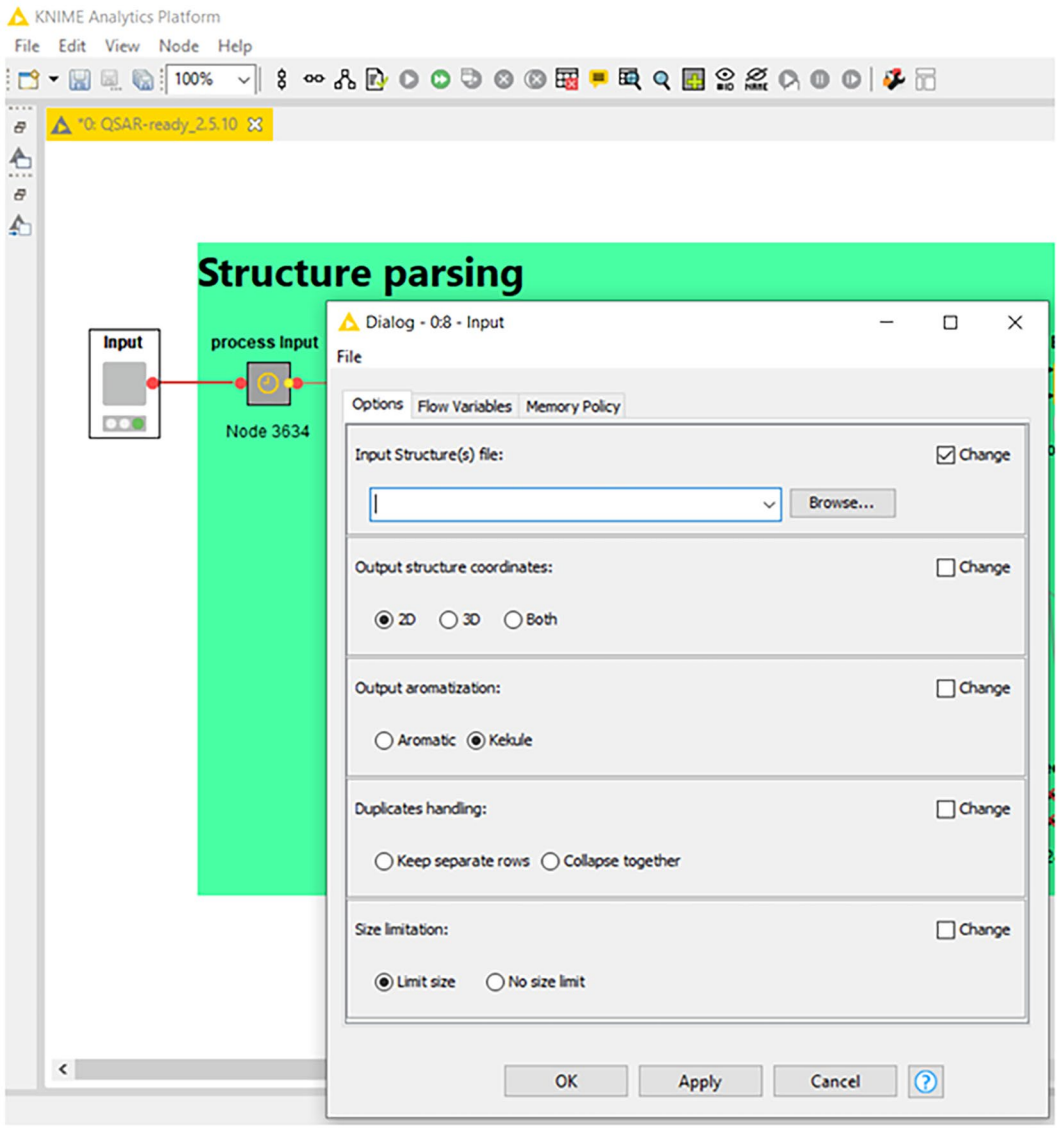
Input and workflow settings

**Fig. 3:**
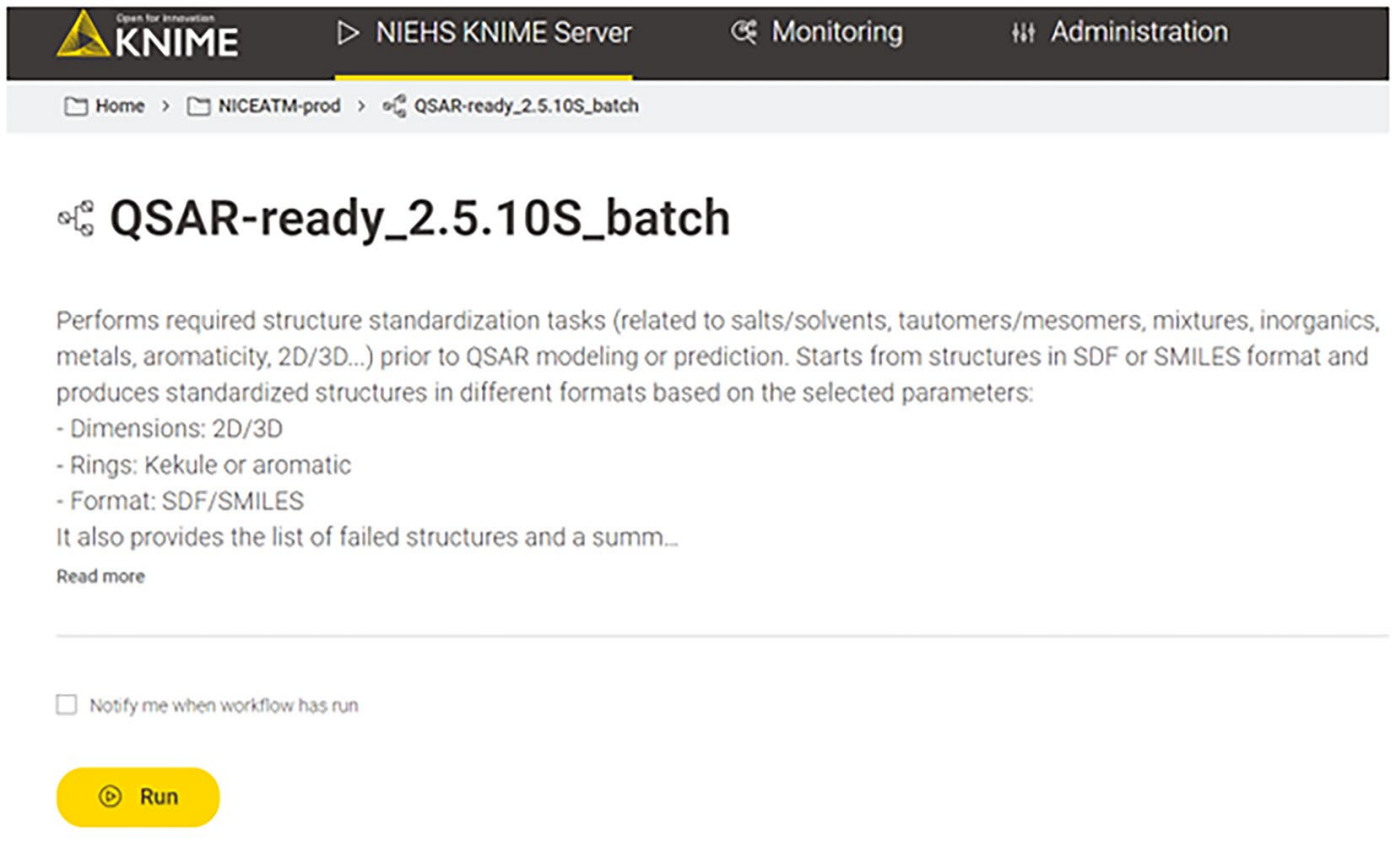
Workflow landing page on the KNIME Server

**Fig. 4:**
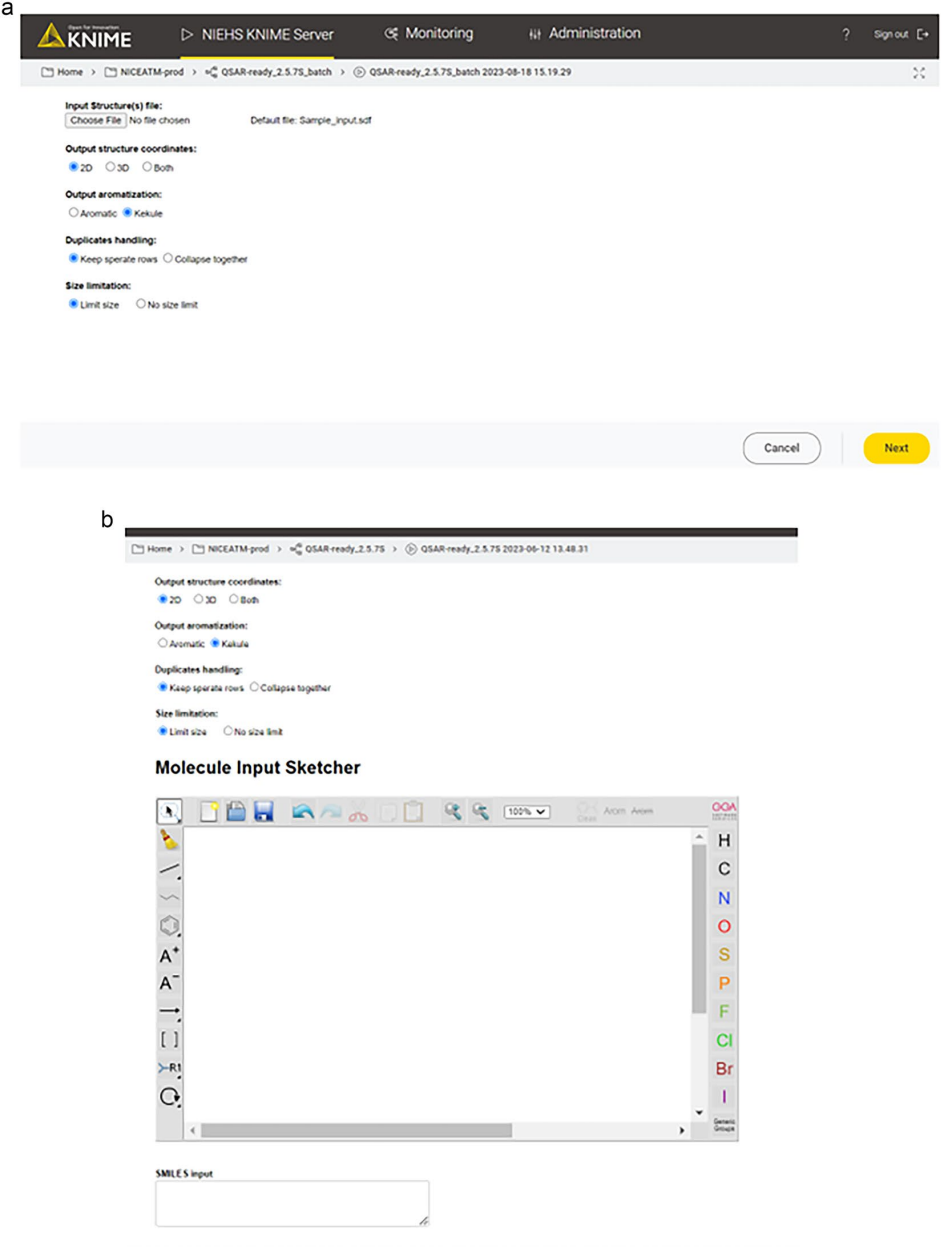
Workflow configuration options on the KNIME Server. **A** File input. **B** Drawing input

This is the most crucial step in running the workflow interactively. In the KNIME environment, the user must double-click on the input component after loading the workflow. This action will open a pop-up window where the user can browse for the input file and configure the workflow parameters, as shown in Fig. 2. The user can then execute the workflow by clicking the green double arrow which automatically configures all the subsequent nodes with the selected parameters as variables. Similarly, when using the WebPortal, the landing page will provide some initial information (Fig. 3), and the user will subsequently be directed to the configuration page (Fig. 4). However, unlike the KNIME environment, on the WebPortal, the user can input structures by pasting SMILES strings or drawing a structure in addition to loading an input file. Currently, the NIEHS KNIME server is only available within the NIH network at https://knime.niehs.nih.gov/knime/. Although running times are hardware dependent, a stress test performed on the NIEHS server revealed an average of 0.017 s/2D structure and 0.038 s/3D structure. The details of the stress test and the server configuration are provided in the Additional file 1: S2.

After the user uploads the input file to be processed, the first step of the workflow will vary based on the file format (MOL, SDF, or SMILES). The workflow will then execute a distinct set of actions to extract the structure information and other pertinent data, including the compound identifiers (CASRN^®^) where applicable. Since SMILES encode chemical structure information in an ASCII string format, they can be either stored in a standard “.smi” text file or in spreadsheets such as “.csv” and Excel files. The workflow supports all these formats except that, in the case of a spreadsheet, the user will be asked to confirm or identify the correct table column containing the structural information and the corresponding unique identifier. After parsing the input file, the workflow will perform a series of initial steps to verify the integrity of the structure(s), check/correct their valence and eliminate any corrupt entries.

When running the workflow in command line, or using the Dockerized version, the user can either leave the default parameters as is or populate the desired variables to configure the workflow differently.

**Fig. 5:**
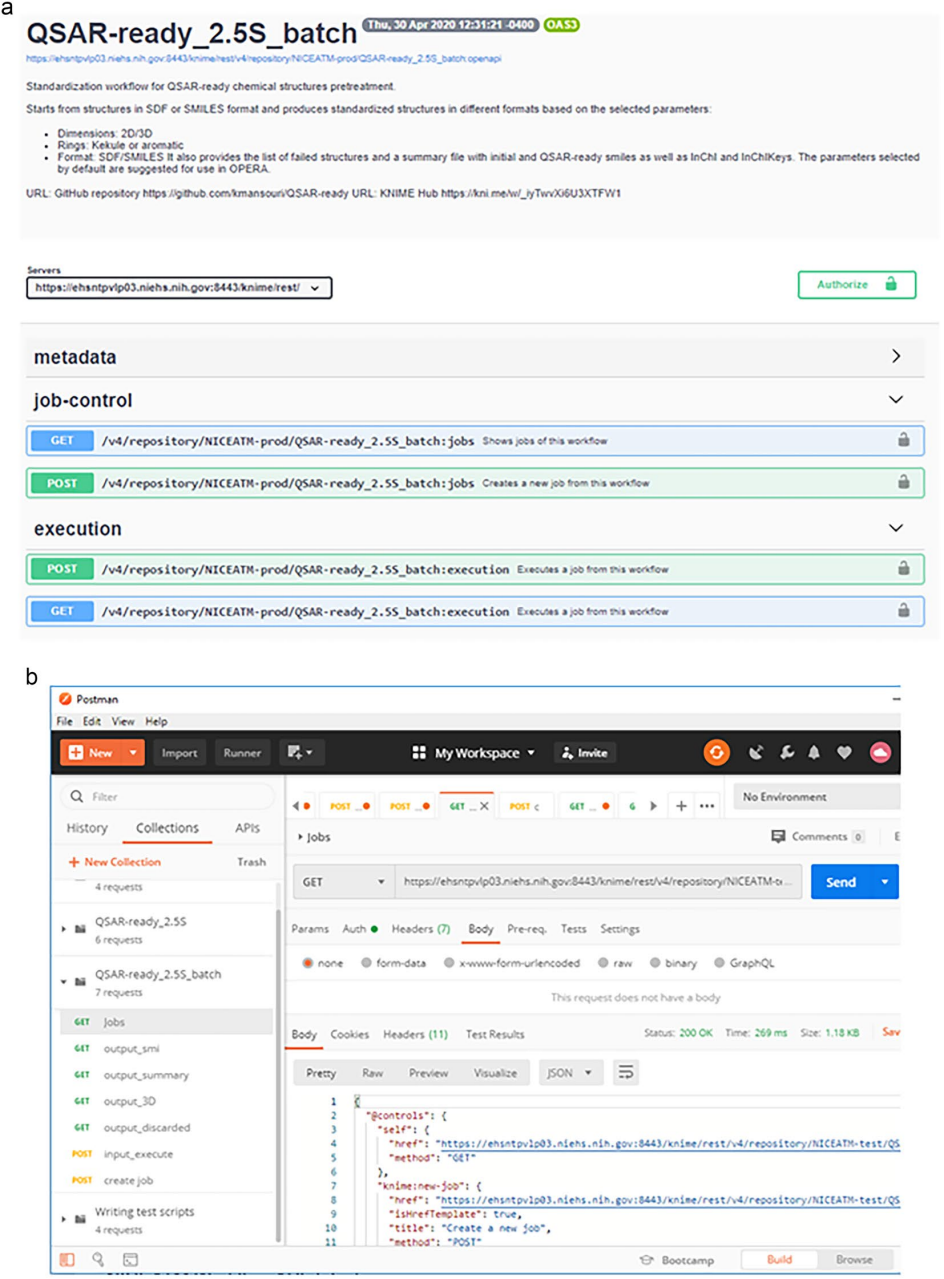
API configuration. **A** On the server. **B** In postman

In addition to the WebPortal, the user can also run the QSAR-ready workflow through the KNIME Server API using a tool such as Postman (Fig. 5). The user needs to launch the Postman application and create a new request. In the new request tab, the user must set the HTTP method (GET, POST, PUT, DELETE) and enter the API URL (https://ehsntpvlp03.niehs.nih.gov:8443/knime/rest/v4/repository/NICEATM-prod/QSAR-ready_2.5S_batch:openapi?showInUI=true) in the address bar. The API currently works only within the NIH network (and will possibly be made public in the future), so the user will need to add authentication in the request headers by navigating to the "Headers" tab. Then, the API request body needs to be populated with the input structure(s) in the appropriate format (e.g., JSON, XML). Once everything is set, the "Send" button will send the API request to the KNIME Server. The server's response, including headers, body, and status code, will be displayed in the "Response" section of the Postman window. The request parameters, headers, or body can be modified as needed and the process repeated by clicking the "Send" button again. It is important to refer to the KNIME Server API documentation for specific details on request formats, authentication methods, and other relevant information for the API (https://www.knime.com/blog/the-knime-server-rest-api).

#### Intermediary steps

**Fig. 6:**
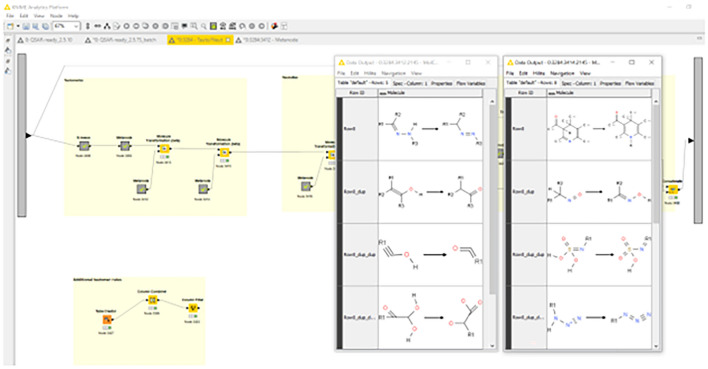
Standardization and neutralization metanode

Once the user clicks on the green double arrow within the KNIME graphical interface, or the “Next” button on the WebPortal, the workflow will automatically execute the above mentioned seven steps. The initial user-selected parameters are translated into variables that are used to configure the “if” statement nodes to go in one direction or another when it comes to choosing between Kekulé and aromatic forms, or whether to deduplicate, filter based on size, or generate 3D structures. The longest step and core of the workflow is the standardization and neutralization metanode. This is performed using a series of transformation rules that are implemented as reactions as depicted in Fig. 6. These transformations follow a specific order so that certain rules do not cancel those that precede them. First, the tautomeric forms are standardized. This includes the nitro- and azide-mesomers, keto-enol, enamine-imine, ynol-ketene tautomers, and other conversions [41, 45, 46]. Additional possible tautomeric rules are also provided in separate nodes for expert users to adapt for specific needs. These transformations are then followed by neutralization of the charged structures, when possible, and removing the stereochemistry information. Explicit hydrogen atoms are then added and structures aromatized according to Hückel’s rule [38, 39, 47].

At each one of these steps, the workflow will carry the processed structures and their related information and will discard the failed structures. However, the latter are not dropped, but rather collected separately with a tag for each one specifying the issue with the structure that led to the failure of the standardization process.

#### Output

At the end of its execution, the workflow generates different files as a result of the process. The standardized QSAR-ready structures are saved in two formats, an SDF file (v2000) and a SMILES file. If the 3D structures were requested, the 3D atom coordinates will be saved in the SDF file. A summary file in CSV format will include the structure identifier, if provided in the input, the original and standardized structures in SMILES format, the standard InChI code and hashed InChI key, as well as the counter ion or salt/solvent, if any. For chemicals with a salt/solvent that was stripped during the process, another CSV file with this information is generated containing an additional generic identifier for the salt or solvent. This information is useful when modeling certain endpoints, where the salt or solvent is relevant. For example, OPERA uses this generic identifier as an additional descriptor for the octanol–water partition coefficient (logKOW), boiling point, melting point, and water-solubility. Any failed structures are collected in a separate CSV file containing the structure identifier (if provided) the original structure and the error that led to the failure of the standardization process. In case the deduplication step is requested by the user, the duplicates will also be provided in the same file with the discarded structures.

**Fig. 7:**
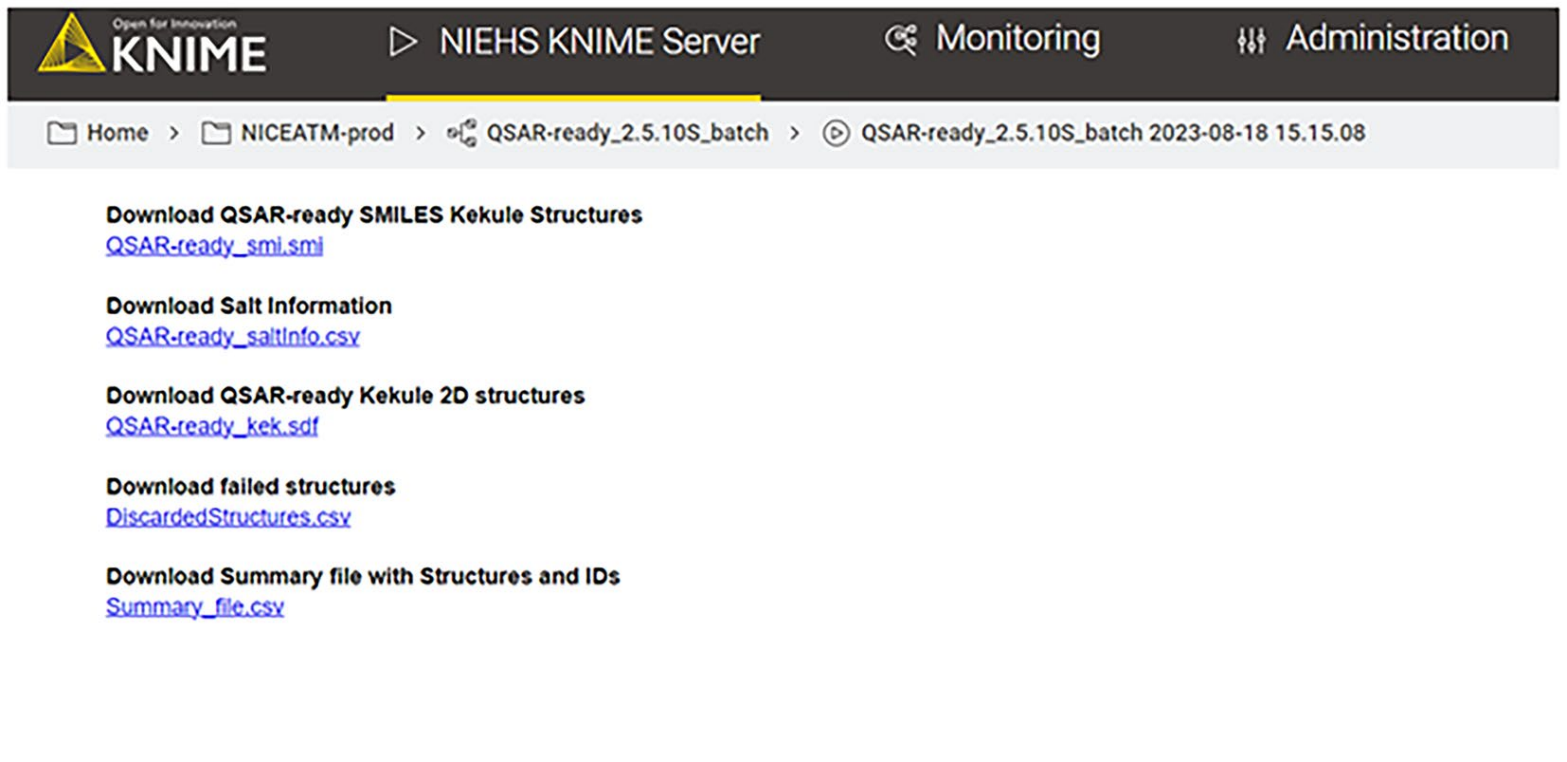
Output files download page

On the WebPortal, the original structures and resulting standardized structures are first displayed for the user prior to the final step where the resulting files are provided for download (Fig. 7). The WebPortal also provides the option to get notified by email when the results are available for download. This option is particularly useful when the input file contains a large number of structures requiring a long time so that the user can even close the browser after launching the workflow. When using the KNIME environment graphical interface or the command line, the output files are written directly to the same folder containing the input file. All resulting files are named using the input file name and adding a specification (such as _Summary_file or _DiscardedStructures) distinguishing the type of information contained in each of the output files. Sample output files are provided on the workflow’s repository page on GitHub: https://github.com/NIEHS/QSAR-ready and examples of standardization transformations are provided in Additional file 3.

Upon the initiation of workflow execution using the KNIME Server API by sending a request with the necessary parameters to the appropriate endpoint, the job progress can be monitored. This monitoring capability facilitates the retrieval of essential job information, including status, logs, and results. Once the job execution is complete, the output results, such as structure data files, can be retrieved through the API endpoints in the desired format. The processed structures can be parsed and analyzed according to the specific requirements. Post-processing actions, such as generating reports, triggering notifications, integrating with other systems, or updating databases, can be performed based on the processed results using the preferred programming language or tools. The KNIME Server API provides a flexible and programmatic means to interact with the KNIME Server and automate various data processing tasks. The official KNIME Server API documentation should be consulted for detailed information on API endpoints, request formats, and available functionalities [43, 44].

### Applications

#### International collaborative modeling projects

This workflow was initially developed for the Collaborative Estrogen Receptor Activity Prediction Project (CERAPP), an international modeling initiative designed to forecast the estrogenic activity of chemicals and organized by the National Center for Computational Toxicology at the U.S. EPA, in support of its Endocrine Disruptor Screening Program (EDSP) [4, 48]. This collaborative project, involving 17 research groups, included chemical structures collected from different public sources that contained many duplicates and inconsistencies. Such issues can alter the molecular descriptor calculation procedure and, subsequently, the quality of the derived QSAR models in terms of accuracy and repeatability. Hence, a structure curation process was required to derive a unique set of QSAR-ready structures that all participating groups could use to have consistent sets of structures for both training and prediction steps. The participants used data from the US ToxCast™ and Tox21 programs [49–51] as a training set to develop and optimize their models. These programs produced concentration–response data for 1812 chemicals, obtained from a collection of 18 in vitro high-throughput screening (HTS) assays that explored multiple sites in the mammalian estrogen receptor (ER) pathway [52]. For the purposes of prioritizing further testing and regulatory measures, we assembled a virtual screening library containing more than 50,000 chemicals identified by their CASRN. This extensive set, gathered from diverse sources, aimed to encompass a substantial proportion of all human-exposed man-made chemicals, and comprised various classes such as consumer products, food additives, and human and veterinary drugs, with significant overlap. Both the training and prediction sets underwent the same structure standardization workflow, resulting in the retention of unique QSAR-ready structures: 1677 in the training set and 32,464 in the prediction set. The participating groups submitted about 50 models using different QSAR and structure-based approaches. These models were then combined into a consensus model that was used to screen the ~ 32 k prediction set.

The CERAPP project demonstrated the effective screening of a large number of environmentally relevant chemicals in a fast and accurate manner by combining multiple modeling approaches in an ensemble method that overcame the limitations of individual models [4]. The project provided valuable data and a screened a list of chemicals. It also served as a successful collaboration example, showcasing the utilization of large amounts of high-quality data in model fitting and rigorous procedures for the development, validation, and use of efficient and accurate methods in predicting human or environmental toxicity while reducing animal testing. The resulting project's workflows including the QSAR-ready standardization were applied to other collaborative modeling projects targeting different toxicological endpoints such as the androgen receptor (AR) activity and acute oral toxicity. The AR modeling project (Collaborative Modeling Project for Androgen Receptor Activity: CoMPARA) adopted the template established by CERAPP and focused on virtually screening chemicals for their potential AR pathway activity based on 11 HTS assays, also in support of the EPA’s EDSP [53]. These assays were conducted on the same initial library of 1855 ToxCast™ chemicals used for ER assays, and the resulting AR pathway activity cores were utilized as a training set in the CoMPARA modeling consortium [25]. A total of 91 qualitative and quantitative predictive QSAR models for binding, agonist, and antagonist AR activities were contributed by collaborators from 25 international research groups. The combined list of chemicals screened by CoMPARA participants using their models included 55,450 chemical structures, comprising both CERAPP chemicals and generated metabolites [54, 55]. The resulting predictions were evaluated using curated literature datasets and subsequently combined into binding, agonist, and antagonist consensus models. The chemical structures from all three datasets for the CoMPARA project (training set, prediction set and evaluation set) were processed using the QSAR-ready standardization workflow prior to modeling and predictions. Both the CERAPP and CoMPARA projects fostered collaboration among participants, aiming to build the best collective consensus rather than competing for the best individual model. Further, the predictions from CERAPP and CoMPARA were accepted by the U.S. EPA as useful for prioritization under the EDSP and for consideration in weight-of-evidence evaluations of endocrine disruption potential [56].

The third crowdsourcing collaborative project that used the QSAR-ready workflow to standardize all used chemical structures was the Collaborative Acute Toxicity Modeling Suite (CATMoS) [26]. Acute systemic toxicity testing is crucial for regulatory hazard classification, labeling, and risk management [57]. However, assessing all new and existing chemicals using traditional rodent acute toxicity tests is time-consuming and expensive. In silico models built using existing data offer a rapid and animal-free approach for predicting acute toxicity. To develop in silico models for predicting acute oral toxicity, the U.S. Interagency Coordinating Committee on the Validation of Alternative Methods (ICCVAM) Acute Toxicity Workgroup organized an international collaboration [58]. The collaboration focused on five different endpoints: Lethal Dose 50 (LD50 value), U.S. Environmental Protection Agency hazard categories (four), Globally Harmonized System for Classification and Labeling hazard categories (five), very toxic chemicals (LD50 ≤ 50 mg/kg), and non-toxic chemicals (LD50 > 2000 mg/kg). An inventory of acute oral toxicity data for 11,992 chemicals was compiled, divided into training and evaluation sets, and shared with 35 participating international research groups. These groups submitted a total of 139 predictive models that were applied on a prediction set that included 48,137 chemical structures, including a hold-out evaluation set of 3000 chemicals. Predictions falling within the applicability domains of the submitted models were evaluated using the external evaluation set and combined to create consensus models that leverage the strengths of individual approaches. The resulting consensus predictions, forming CATMoS, exhibit high accuracy and robustness when compared to in vivo results [26, 59]. Regulatory agencies are currently evaluating CATMoS for its utility and applicability as a potential replacement for in vivo rat acute oral toxicity studies.

These successful collaborative projects support international cooperation, exemplify the collaborative resolution of toxicological challenges through computational approaches, establish a legacy of freely available open-source code and workflows, and contribute to the growing recognition and acceptance of non-animal methods by regulatory authorities.

#### OPERA models

CERAPP, CoMPARA and CATMoS consensus models were implemented in OPERA, a free and open source/open data suite of over twenty QSAR models. OPERA models possess the capability to assess chemicals for toxicity endpoints, as well as predict physicochemical, environmental fate, and ADME properties. These models adhere to the five OECD principles for QSAR modeling, ensuring scientific validity and high accuracy while minimizing complexity [60]. The models are built on thoroughly curated experimental data and standardized chemical structures using the QSAR-ready workflow. The workflow is also embedded in OPERA so that input chemical structures are standardized in a manner consistent with the training sets of the models prior to prediction.

In the latest version, several OPERA models, including physicochemical properties and ADME parameters, have been updated with the latest publicly available datasets. These updates enhance predictivity, expand the applicability domain coverage, and consider extensively studied groups of chemicals like polyfluorinated substances (PFAS). The suite allows predictions to be generated for individual chemicals or in batch mode, and input chemical structures can be processed using OPERA's internal QSAR-ready standardization workflow or provided via structure identifiers from EPA's DSSTox database, which comprises over 1 million curated chemical structures [23, 61].

OPERA provides comprehensive prediction reports that include accuracy estimates, applicability domain assessments, confidence ranges, and, whenever possible, experimental values. Technical and performance details are presented in OECD-compliant QSAR model reporting format (QMRF) reports. The predictions generated by OPERA on the whole DSSTox database can be accessed through EPA's CompTox Chemicals Dashboard, the National Toxicology Program's (NTP) Integrated Chemical Environment (ICE), and recently through FDA's Precision Platform [24, 27, 62].

OPERA can be downloaded as a standalone command-line or graphical user interface compatible with Windows and Linux operating systems from the NIEHS GitHub repository (https://github.com/NIEHS/OPERA). It can also be used as a plugin within the OECD's QSAR Toolbox (https://repository.qsartoolbox.org/Tools/Details/6703ab01-9529-4f86-814f-6efc49e1f59c) and is available as Python, C/C++, and Java libraries that can be integrated into other applications.

#### Modeling and database search

The standardization workflow was used to generate QSAR-ready structures for the over 1 M chemicals of the EPA’s DSSTox database. These structures were made available for single and batch mode download on the EPA’s CompTox Chemicals Dashboard as well as through its API webservice. Consequently, the standardized structures are being used for different levels of mappings and facilitate searching of related compounds on both the EPA’s dashboard and NTP’s ICE dashboard [24, 27].

Additionally, the online availability of this large number of QSAR-ready structures, linked to their related chemical identifiers (CASRN, DTXSID, names), facilitated the progress on several other projects and modeling studies such as NTA [22, 63]. Chemical database searching has become an integral part of non-targeted identification workflows using HRMS. However, the observed form of a chemical structure in HRMS may not always match the form stored in a database, such as the neutral form versus a salt or a component of a mixture rather than the mixture form found in consumer products. To address this, a KNIME workflow has been developed to link the observed HRMS structures ("MS-Ready structures") to their corresponding forms stored in a database [22]. These MS-Ready structures, along with their mappings to full chemical representations, are accessible through the US EPA's CompTox Chemicals Dashboard.

To establish connections between specific forms of a substance and their structure components (e.g., salts and mixtures) as well as their related MS-Ready forms, structure standardization is necessary. Since the requirements for MS-Ready structures are similar, and due to the flexibility of KNIME workflows, the previously developed QSAR-Ready workflow was modified to produce MS-Ready chemical structure forms suitable for database searching. The resulting KNIME workflow, rule set, and software processing module for generating MS-Ready structures are publicly available for download from GitHub (https://github.com/kmansouri/MS-ready).

Furthermore, the adapted workflow was used to generate MS-Ready forms for all chemicals in the DSSTox database, enabling access via the US EPA's CompTox Chemicals Dashboard. The Dashboard provides functionalities such as searching, exporting, and downloading MS-Ready structures. The value of MS-Ready structures is demonstrated through several examples, including their integration with the in silico fragmenter MetFrag for identification in NTA [64]. By offering access to MS-Ready structures and facilitating integration with MetFrag, these resources contribute to the structural identification of chemicals, including mixtures and salts, for the wider scientific community.

Currently, the EPA is in process of replicating the QSAR-ready and MS-ready standardization workflows to fully embed and integrate them in the chemical curation and registration process of DSSTox database. This will not only benefit the different mappings and search functionalities of the dashboard, but also ongoing and future modeling and NTA studies.

### Limitations and improvements

KNIME is a popular open-source data analytics platform that provides a wide range of functionalities for data processing, modeling, and visualization. While KNIME is a versatile and flexible platform, it does have some limitations that users should be aware of. One of the most significant limitations is its steep learning curve, which can be challenging for users new to data analytics or programming. KNIME's extensive range of features and capabilities can be overwhelming, and users may require significant time and effort to master the platform's functionalities. Additionally, KNIME can be resource-intensive, depending on the size of the datasets and complexity of the workflows. Users may need to invest in high-performance hardware or cloud-based solutions to run workflows efficiently. While KNIME's visualization capabilities are not as advanced as some other data analytics tools, its compatibility with other software solutions makes it a valuable resource for researchers and data scientists. KNIME can integrate with various data visualization tools, such as Tableau and Qlik, allowing users to create more advanced visualizations [65, 66]. While KNIME does offer a wide range of machine learning algorithms, some users may find that the platform's machine learning libraries are limited compared to other tools. Users who require access to more advanced machine learning algorithms may need to use other software solutions in combination with KNIME.

Overall, while KNIME has some limitations, its flexibility, versatility, and compatibility with other tools make it a valuable resource for researchers and data scientists working in various domains. Also, most of the limitations related only concern workflow developers. Once at the expert level, the developers can simplify running their workflows for users by either adding a user-friendly configuration window using the graphical interface or packaging the workflow within another application. These two options were provided to the users of the QSAR-ready workflow as mentioned above on the one-step configuration window and running the workflow within OPERA without requiring installation of KNIME.

## Conclusion

The QSAR-ready chemical structure standardization workflow in KNIME offers a wide range of features and capabilities that make it an ideal choice for different cheminformatic applications. With its flexible and extendable nature, the workflow can be used for a variety of modeling projects and collaborations, providing users with the ability to work on different tasks within the same platform.

KNIME offers a stable and backward-compatible platform for QSAR-related chemical structure standardization. This stability ensures that old nodes are deprecated rather than removed, allowing previous workflows to be reliably executed even with newer versions. This facilitates smooth software upgrades without workflow disruptions. Additionally, KNIME's standalone version seamlessly integrates with tools like OPERA, the Dashboard, and ICE, expanding its functionality. This integration enables users to perform various tasks within a unified environment. KNIME boasts a user-friendly interface with drag-and-drop features for convenient data interaction.

Thanks to KNIME flexibility, the QSAR-ready chemical structure standardization workflow is designed to handle various data formats and is easily scalable and expandable to meet different cheminformatic needs beyond QSARs (such as database search and exploration, similarity search, read-across…). The platform offers a wide range of functionalities, including data preprocessing, feature selection, model training, and evaluation, all of which can be easily customized to suit the user's needs.

Overall, the QSAR-ready chemical structure standardization workflow in KNIME provides a comprehensive and reliable solution for researchers and data scientists working in the field of QSAR. Its flexibility, stability, and backward compatibility, as well as its seamless integration with other tools, make it an invaluable resource for anyone seeking an efficient and reliable platform for their data analytics work.

### Supplementary Information

**Additional file 1:S1.** Example of QSAR standardization rule non-commutation. S2: Stress test of the workflow onthe NIEHS KNIME Server.

**Additional file 2:S2.** Rules applied in the standardization process.

**Additional file 3.S3.** Examples of standardization transformations.

## Data Availability

The QSAR-ready workflow is available on the KNIME Hub at: https://kni.me/w/5TRvnGfMJsgTkcZu. Users can also share KNIME workflows through GitHub, a web-based platform for software version control and collaboration. The QSAR-ready workflow is available on the NIEHS GitHub repository: https://github.com/NIEHS/QSAR-ready. Another way to share KNIME workflows is through Docker containers. By creating a container image that includes the KNIME workflow and all the dependencies required to run it, users can share their workflows with others and run them on any machine that supports Docker, regardless of the underlying operating system or hardware. The QSAR-ready workflow is available as a docker container at: https://hub.docker.com/r/kamelmansouri/qsar-ready. The docker file is available on GitHub at: https://github.com/kmansouri/QSAR-ready which is built on a KNIME base image developed by: https://github.com/ibisba/knime-workflow-base. Finally, KNIME Server provides a web-based platform for centralized management of workflows, data, and users. By deploying their workflows to the server, users can share them with other users in their organization, run them remotely, schedule them to run at specific times, and monitor their execution. The QSAR-ready workflow is available on NIEHS’ KNIME server WebPortal at: https://knime.niehs.nih.gov/knime/.

## References

[1] Fourches D, Muratov E, Tropsha A (2016). Trust, but verify II: a practical guide to chemogenomics data curation. J Chem Inf Model.

[2] Alex B, Grover C, Haddow B (2008). Automating curation using a natural language processing pipeline. Genome Biol.

[3] Cao D, Liang Y, Xu Q (2011). Toward better QSAR/QSPR modeling: simultaneous outlier detection and variable selection using distribution of model features. J Comput Aided Mol Des.

[4] Mansouri K, Abdelaziz A, Rybacka A (2016). CERAPP: collaborative estrogen receptor activity prediction project. Environ Health Perspect.

[5] Mansouri K, Grulke CM, Richard AM (2016). An automated curation procedure for addressing chemical errors and inconsistencies in public datasets used in QSAR modelling. SAR QSAR Environ Res.

[6] Fourches D, Muratov E, Tropsha A (2010). Trust, but verify: on the importance of chemical structure curation in cheminformatics and QSAR modeling research. J Chem Inf Model.

[7] Williams AJ, Ekins S, Tkachenko V (2012). Towards a gold standard: regarding quality in public domain chemistry databases and approaches to improving the situation. Drug Discov Today.

[8] Williams AJ, Ekins S (2011). A quality alert and call for improved curation of public chemistry databases. Drug Discov Today.

[9] Lowe CN, Charest N, Ramsland C (2023). Transparency in modeling through careful application of OECD’s QSAR/QSPR principles via a curated water solubility data set. Chem Res Toxicol.

[10] Young D, Martin T, Venkatapathy R, Harten P (2008). Are the chemical structures in your QSAR correct?. QSAR Comb Sci.

[11] Karapetyan K, Batchelor C, Sharpe D (2015). The chemical validation and standardization platform (CVSP): large-scale automated validation of chemical structure datasets. J Cheminformatics.

[12] Bento AP, Hersey A, Felix E (2020). An open source chemical structure curation pipeline using RDKit. J Cheminform.

[13] Cretu MT, Toniato A, Thakkar A, Debabeche A, Laino T, Vaucher AC (2023) Standardizing chemical compounds with language models. ChemRxiv. 10.26434/chemrxiv-2022-14ztf-v2

[14] Hähnke VD, Kim S, Bolton EE (2018). PubChem chemical structure standardization. J Cheminform.

[15] Swain M (2023) MolVS: molecule validation and standardization. https://github.com/mcs07/MolVS. Accessed 8 Feb 2023

[16] MolVS: molecule validation and standardization—MolVS 0.1.1 documentation. https://molvs.readthedocs.io/en/latest/. Accessed 11 Jan 2023

[17] Dolciami D, Villasclaras-Fernandez E, Kannas C (2022). CanSAR chemistry registration and standardization pipeline. J Cheminform.

[18] Jeliazkova N, Kochev N, Jeliazkov V (2016) Ambitcli-3.0.2. https://zenodo.org/records/173560

[19] Berthold MR, Cebron N, Dill F et al (2008) KNIME: the konstanz information miner. In: Preisach C, Burkhardt H, Schmidt-Thieme L, Decker R (eds) Data analysis, machine learning and applications: proceedings of the 31st annual conference of the Gesellschaft für Klassifikation e.V., Albert-Ludwigs-Universität Freiburg, March 7–9, 2007. Springer, Berlin, pp 319–326

[20] Mansouri K (2016) OPERA: Command line application providing QSAR models predictions as well as applicability domain and accuracy assessment. Software GitHub repository. https://github.com/kmansouri/OPERA.

[21] Mansouri K, Grulke CM, Judson RS, Williams AJ (2018). OPERA models for predicting physicochemical properties and environmental fate endpoints. J Cheminform.

[22] McEachran AD, Mansouri K, Grulke C (2018). “MS-Ready” structures for non-targeted high-resolution mass spectrometry screening studies. J Cheminform.

[23] Grulke CM, Williams AJ, Thillanadarajah I, Richard AM (2019). EPA’s DSSTox database: history of development of a curated chemistry resource supporting computational toxicology research. Comput Toxicol.

[24] Williams AJ, Grulke CM, Edwards J (2017). The CompTox chemistry dashboard: a community data resource for environmental chemistry. J Cheminform.

[25] Mansouri K, Nicole K, Abdelaziz AM (2020). CoMPARA: collaborative modeling project for androgen receptor activity. Environ Health Perspect.

[26] Mansouri K, Karmaus AL, Fitzpatrick J (2021). CATMoS: collaborative acute toxicity modeling suite. Environ Health Perspect.

[27] Bell S, Abedini J, Ceger P (2020). An integrated chemical environment with tools for chemical safety testing. Toxicol Vitro Int J Publ Assoc BIBRA.

[28] Lowe CN, Williams AJ (2021). Enabling high-throughput searches for multiple chemical data using the US-EPA CompTox chemicals dashboard. J Chem Inf Model.

[29] Kolmar SS, Grulke CM (2021). The effect of noise on the predictive limit of QSAR models. J Cheminform.

[30] Bengio Y, Courville A, Vincent P (2013). Representation learning: a review and new perspectives. IEEE Trans Pattern Anal Mach Intell.

[31] Waldo WH (1962). Searching two-dimensional structures by computer. J Chem Doc.

[32] Apodaca RL (2020) A guide to molecular standardization. http://depth-first.com/articles/2020/07/27/a-guide-to-molecular-standardization/. Accessed 11 Jan 2023

[33] Anderson E, Veith G, Weininger D (1987) SMILES: a line notation and computerized interpreter for chemical structures. https://api.semanticscholar.org/CorpusID:64884759

[34] Dalby A, Nourse JG, Hounshell WD (1992). Description of several chemical structure file formats used by computer programs developed at molecular design limited. J Chem Inf Comput Sci.

[35] James CA, Weininger D, Delany J (2008) Daylight theory manual. Chemical information systems, Aliso Viejo, CA, USA

[36] Dassault Systèmes (2020) CTfile formats. In: Dassault systèmes. https://discover.3ds.com/ctfile-documentation-request-form. Accessed 17 Aug 2023

[37] Baker CM, Kidley NJ, Papachristos K (2020). Tautomer standardization in chemical databases: deriving business rules from quantum chemistry. J Chem Inf Model.

[38] Hückel E (1932). Quantentheoretische beiträge zum benzolproblem. III. Quantentheoretische beiträge zumproblemder aromatischenundungesättingten verbindungen. Z Phys Ger.

[39] Kekulé A (1866). Untersuchungen über aromatische Verbindungen. Liebigs Ann Chem.

[40] Dhaked DK, Ihlenfeldt W-D, Patel H (2020). Toward a comprehensive treatment of tautomerism in chemoinformatics including in InChI V2. J Chem Inf Model.

[41] Sitzmann M, Ihlenfeldt W-D, Nicklaus MC (2010). Tautomerism in large databases. J Comput Aided Mol Des.

[42] Fillbrunn A, Dietz C, Pfeuffer J (2017). KNIME for reproducible cross-domain analysis of life science data. J Biotechnol.

[43] KNIME Server User Guide. https://docs.knime.com/latest/server_user_guide/index.html#introduction. Accessed 16 May 2023

[44] The KNIME Server REST API. In: KNIME. https://www.knime.com/blog/the-knime-server-rest-api. Accessed 16 May 2023

[45] ChemAxon (2014) ChemAxon Standardizer–Cheminformatics platforms and desktop applications. http://www.chemaxon.com/products/standardizer/. Accessed 25 Nov 2014

[46] Reusch W (2013) Examples of chemical reactions. http://www2.chemistry.msu.edu/faculty/reusch/virttxtjml/react2.htm. Accessed 25 Nov 2014

[47] von E. Doering W, Detert FL (1951). Cycloheptatrienylium oxide. J Am Chem Soc.

[48] US EPA OCSPP (2023) EPA rebuilds endocrine disruptor screening program by soliciting public comment on new approach methodologies to screen for endocrine effects. https://www.epa.gov/pesticides/epa-rebuilds-endocrine-disruptor-screening-program-soliciting-public-comment-new. Accessed 3 May 2023

[49] Dix DJ, Houck KA, Martin MT (2007). The ToxCast program for prioritizing toxicity testing of environmental chemicals. Toxicol Sci.

[50] Huang R, Sakamuru S, Martin MT (2014). Profiling of the Tox21 10K compound library for agonists and antagonists of the estrogen receptor alpha signaling pathway. Sci Rep.

[51] Judson RS, Houck KA, Kavlock RJ (2010). In vitro screening of environmental chemicals for targeted testing prioritization: the ToxCast project. Environ Health Perspect.

[52] Judson RS, Magpantay FM, Chickarmane V (2015). Integrated model of chemical perturbations of a biological pathway using 18 in vitro high-throughput screening assays for the estrogen receptor. Toxicol Sci.

[53] Kleinstreuer NC, Ceger P, Watt ED (2017). Development and validation of a computational model for androgen receptor activity. Chem Res Toxicol.

[54] Leonard JA, Stevens C, Mansouri K (2018). A workflow for identifying metabolically active chemicals to complement in vitro toxicity screening. Comput Toxicol.

[55] Pinto CL, Mansouri K, Judson R, Browne P (2016). Prediction of estrogenic bioactivity of environmental chemical metabolites. Chem Res Toxicol.

[56] US EPA (2023) Availability of new approach methodologies (NAMs) in the endocrine disruptor screening program (EDSP). https://www.regulations.gov/document/EPA-HQ-OPP-2021-0756-0002. Accessed 31 July 2023

[57] Strickland J, Clippinger AJ, Brown J (2018). Status of acute systemic toxicity testing requirements and data uses by U.S. regulatory agencies. Regul Toxicol Pharmacol.

[58] Kleinstreuer NC, Karmaus AL, Mansouri K (2018). Predictive models for acute oral systemic toxicity: a workshop to bridge the gap from research to regulation. Comput Toxicol.

[59] Karmaus AL, Mansouri K, To KT (2022). Evaluation of variability across rat acute oral systemic toxicity studies. Toxicol Sci Off J Soc Toxicol.

[60] OECD (2007) Guidance document on the validation of (quantitative) structure–activity relationship [(Q)SAR] models. Guid doc valid quant struct-act relatsh QSAR models

[61] Richard AM, Williams CR (2002). Distributed structure-searchable toxicity (DSSTox) public database network: a proposal. Mutat Res.

[62] PrecisionFDA—overview. https://precision.fda.gov/. Accessed 16 May 2023

[63] Sobus JR, Wambaugh JF, Isaacs KK (2018). Integrating tools for non-targeted analysis research and chemical safety evaluations at the US EPA. J Expo Sci Environ Epidemiol.

[64] Ruttkies C, Schymanski EL, Wolf S (2016). MetFrag relaunched: incorporating strategies beyond in silico fragmentation. J Cheminform.

[65] Business Intelligence and Analytics Software | Tableau. https://www.tableau.com/. Accessed 2 Feb 2024

[66] Qlik Data Integration, Data Quality, and Analytics Solutions. In: Qlik. https://www.qlik.com/us. Accessed 2 Feb 2024

