# Supplementary material for: Free and open-source QSAR-ready workflow for automated standardization of chemical structures in support of QSAR modeling
Source: J Cheminform. 2024 Feb 20;16:19. doi: 10.1186/s13321-024-00814-3 (PMC10880251; doi:10.1186/s13321-024-00814-3)
Supplement: Supplementary file 1 — Additional file 1:S1. Example of QSAR standardization rule non-commutation. S2: Stress test of the workflow onthe NIEHS KNIME Server. [file 13321_2024_814_MOESM1_ESM.docx]

S1: Example of QSAR standardization rule non-commutation


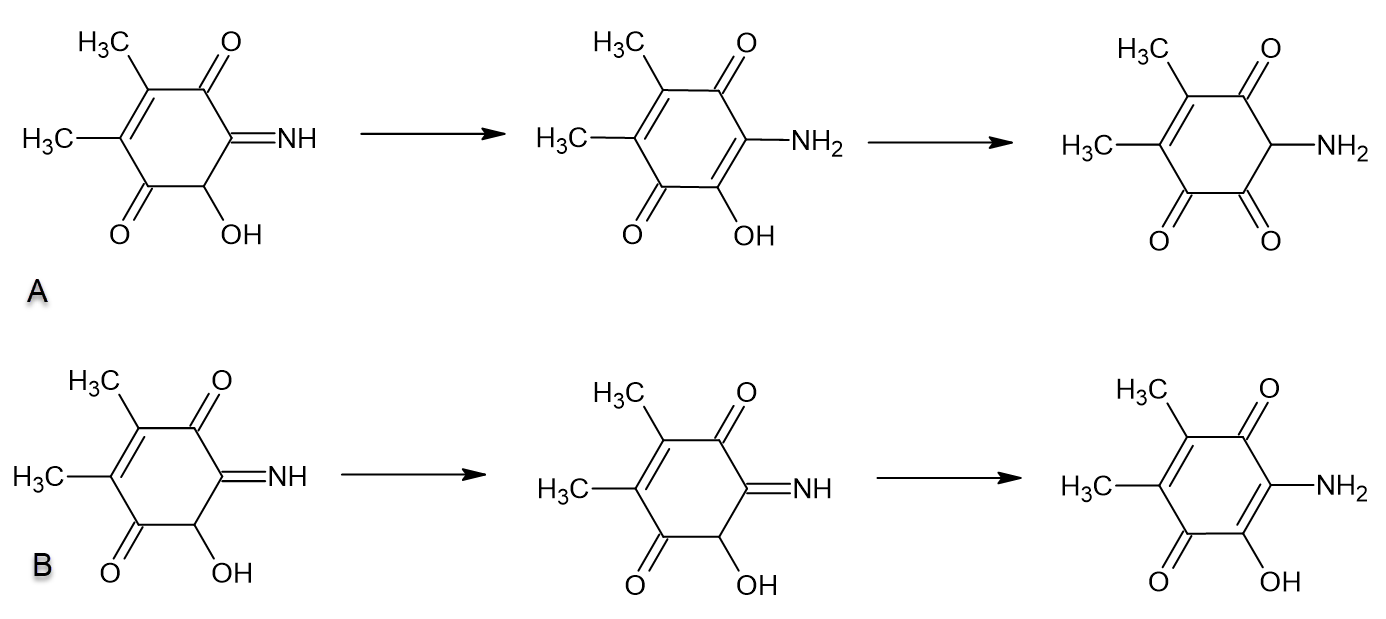


Consider the structure for 5-hydroxy-6-imino-2,3-dimethylcyclohex-2-ene-1,4-dione, N=C1C(O)C(=O)C(C)=C(C)C1=O. As imaged in Figure x.A, suppose if this chemical is standardized first to the amine form of the molecule using an imine to amine rule, the result would be, 2-amino-3-hydroxy-5,6-dimethylcyclohexa-2,5-diene-1,4-dione, C1(C)=C(C(=O)C(N)=C(C1=O)O)C. Next, a keto-enol rule is applied to transform the molecule into the enol form of the molecule, 3-amino-5,6-dimethylcyclohex-5-ene-1,2,4-trione, NC1C(=O)C(=O)C(C)=C(C)C1=O. As shown in Figure x.B, this would different if the keto-enol rule was applied first, which would not result in a transformation of 5-hydroxy-6-imino-2,3-dimethylcyclohex-2-ene-1,4-dione, N=C1C(O)C(=O)C(C)=C(C)C1=O. The imine-amine rule being applied second would then result in a final structure for 2-amino-3-hydroxy-5,6-dimethylcyclohexa-2,5-diene-1,4-dione, NC1=C(O)C(=O)C(C)=C(C)C1=O, not incorporating the keto-enol rule change.

S2: Stress test on the NIEHS KNIME Server (v. 4.15.3) running on CentOS Linux 3.10.0. CPU - Intel(R) Xeon(R) Gold 6226 CPU @ 2.70GHz. RAM - 96 GiB

| File | No. of compounds | File Size (MB) | 2D/3D structures | Total Time (s) | Time per Compound (s) |
| --- | --- | --- | --- | --- | --- |
| Phenotypic Screening Library | 5,760 | 14.8 | 2D | 109 | 0.019 |
| Covalent Screening Library | 11,760 | 14.45 | 2D | 192 | 0.016 |
| Discovery Diversity Set | 50,240 | 72.03 | 2D | 820 | 0.016 |
| Phenotypic Screening Library | 5,760 | 14.8 | 3D | 231 | 0.040 |
| Covalent Screening Library | 11,760 | 14.45 | 3D | 440 | 0.037 |
| Discovery Diversity Set | 50,240 | 72.03 | 3D | 1890 | 0.038 |
